# 

**Published:** 1862-02

**Authors:** 


### CONTENTS.---FEBRUARY, 1862.

### ORIGINAL CONTRIBUTIONS.

PAGE

ART. V. OVARIAN TUMORS. By WM. H. BYFORD, A. M., M. D., Professor of Obstetrics and Diseases of Women and Children, in the Medical Department, Lind University, Chicago, Ill.,

3d Paper :—Causes and Prognosis of Ovarian Dropsy, .......... 65

4th Paper :—Diagnosis of Ovarian Dropsy, .......... 70

ART. VI. Report on the Prevalent Diseases of the City of Chicago, from Nov. 1st, 1861, to February 1st, 1862, including two cases of Puerperal Convulsions. By N. S. DAVIS, M. D., Member of the Sanitary Committee, .......... 88

ART. VII. The Use of Veratrum Viride as an Arterial Sedative, first discovered by a Veterinary Surgeon, .......... 96

### SELECTIONS.

Homœopathy in Military Hospitals, .......... 97

Chloroform or Ether, .......... 104

Uterine Hæmatocele, .......... 107

### BOOK NOTICES.

A System of Surgery: Pathological, Diagnostic, Therapeutic and Operative. By SAMUEL D. GROSS, M. D., Prof. of Surgery in the Jefferson Medical College, etc. etc., .......... 111

Vaiedictory to the Graduates of Rush Medical College, February 5th, 1862. By J. W. FREER, M. D., Prof. of Physiology and Surgical Anatomy, .......... 114

Religio Medici, A Letter to a Friend, Christian Morals, Urn Burial, and other Papers. By Sir THOMAS BROWNE, Kt., M. D., .......... 115

### EDITORIAL.

American Medical Association, .......... 116

Summer Course of Medical Instruction in this City, .......... 117

Illinois State Medical Society, .......... 118

The Wounded at Fort Donelson—Letter from the Battle-Field, .......... 119

Medical Department of Lind University—Annual Commencement, .......... 124

Obituary—Dr. J. N. GRAHAM., .......... 125

Honor to AGASSIZ, .......... 125

SPECIAL SELECTIONS :—Propylamin, .......... 125

Stimulants in Military Hospitals, .......... 126

Restoration of Bone, .......... 127

